# Gunshot Fracture of Clavicle

**Published:** 1897-02

**Authors:** R. B. Longmire

**Affiliations:** Jacksonville, Texas


For the Texas Medical Journal.

### GUNSHOT FRACTURE OF CLAVICLE — NOTES OF CASE.

BY R. B. LONGMIRE, M. D., JACKSONVILLE, TEXAS.

[Read at East Texas Medical Society, Jacksonville, January 12, 1897.]

ON THE 25th of November, 1894, P. H., a mulatto, aged about fifteen, was shot accidentally. The load (of squirrel shot) took effect on the right side, at a point midway between the nipple and the middle of the clavicle, and, ranging upward, struck the clavicle near the middle, producing a splintery fracture of the bone at that point. The load carried away all the tissues, including about two inches of the bone and periosteum, which were in its track. The force of the powder detached the periosteum from the anterior and inferior surfaces of the inner fragment as far as the joint, and the outer end of this fragment was driven upward into the neck. The orifice of entrance was something larger than a half dollar, and that of exit was about the size of the palm of the hand. From the entrance to the clavicle, the track appeared very much like an auger-hole, being smooth and standing open; and from a level with the lower border of the clavicle to the surface above, the wound spread out and was somewhat funnel-shaped, being clean-cut and smooth, with the exception of a number of splinters of bone from one-half to two inches long, which were sticking in the soft tissues. The right arm was pulseless, cold and numb from the shoulder down. The skin around the entrance was powder-burnt, and the entire surface of the wound, including the fissures in the bone, was blackened by the powder.

The inner fragment of the clavicle was split, splintered, and so badly damaged, we decided to remove it. An incision was made from the inner margin of the wound, along the natural site of this part of the clavicle, to a little beyond the joint. The periosteum was carefully separated from the superior and posterior surfaces, and the fragment disarticulated and removed. The splintered end of the outer fragment was sawed off, the splinters of bone removed from the soft tissues, and the lower portion of the wound laid open. The wound was then thoroughly cleansed, first, with sterilized water, and then with bichloride solution 1-2000, the surface dried, dusted with iodoform, and packed with iodoform gauze. There was so much loss of tissue, the wound could not be closed. After the operation was done, and dressings applied so as to raise the outer fragment of the clavicle, the right arm regained its circulation, warmth and sensation.

The dressings were changed every day for a few days, and afterwards every other day. The discharge from the wound resembled synovial fluid, and was very free for several days. There was no pus, no fever, at any time. The wound healed by healthy granulation from start to finish.

Five weeks after he was wounded, the lad began to do light work, and during the year 1895 he made a regular hand on the farm, having good use of his right arm. About ten months after the operation, I saw him, examined the site of the wound, and found that a *new bone* had formed in the place, and about the size and length of the fragment destroyed and removed, forming a joint at the sternum and being firmly united to the inner end of the outer fragment, thus making a clavicle, to all intents and purposes, as good as the old one. The new bone was not curved as the old one.

This case is unique, to the writer, on account of a new bone forming in an open wound, which was packed from time to time throughout the entire treatment, and which healed by granulation from beginning to end; the bone being removed, two inches of which, together with its periosteum, being blown away. I noticed during the process of healing a yellowish gelatinous substance extending along the site of the old clavicle, at bottom of the wound, which was finally covered in by the granulations forming the soft tissues.

